# Photothermal Effects and Heat Conduction in Nanogranular Silicon Films

**DOI:** 10.3390/nano11092379

**Published:** 2021-09-13

**Authors:** Bayan A. Kurbanova, Gauhar K. Mussabek, Viktor Y. Timoshenko, Vladimir Lysenko, Zhandos N. Utegulov

**Affiliations:** 1 Department of Physics, School of Sciences and Humanities, Nazarbayev University, Nur-Sultan 010000, Kazakhstan; bayan.kurbanova@nu.edu.kz; 2 Faculty of Physics and Technology, Al-Farabi Kazakh National University, Almaty 050040, Kazakhstan; gauhar.musabek@kaznu.kz; 3 Laboratory “Nanotheranostics”, Institute of Engineering Physics for Biomedicine, National Research Nuclear University “MEPhI”, 115409 Moscow, Russia; victor_timoshenk@mail.ru (V.Y.T.); vladimir.lysenko@univ-lyon1.fr (V.L.); 4 Faculty of Physics, Lomonosov Moscow State University, 119911 Moscow, Russia; 5 Lebedev Physical Institute of RAS, 119911 Moscow, Russia; 6 Light Matter Institute, UMR-5306, Claude Bernard University of Lyon, 2 rue Victor Grignard, 69622 Villeurbanne, France

**Keywords:** silicon, nanogranular, nanoparticle, nanostructure, porous, void, drop casting, thin film, laser heating, photothermal, temperature, Raman, phonons, heat conduction, phase transition, finite element modeling, thermal conductivity, FDTD, FEM, phase transition

## Abstract

We present results on the photothermal (PT) and heat conductive properties of nanogranular silicon (Si) films synthesized by evaporation of colloidal droplets (drop-casting) of 100 ± 50 nm-sized crystalline Si nanoparticles (NP) deposited on glass substrates. Simulations of the absorbed light intensity and photo-induced temperature distribution across the Si NP films were carried out by using the Finite difference time domain (FDTD) and finite element mesh (FEM) modeling and the obtained data were compared with the local temperatures measured by micro-Raman spectroscopy and then was used for determining the heat conductivities k in the films of various thicknesses. The cubic-to-hexagonal phase transition in Si NP films caused by laser-induced heating was found to be heavily influenced by the film thickness and heat-conductive properties of glass substrate, on which the films were deposited. The k values in drop-casted Si nanogranular films were found to be in the range of lowest k of other types of nanostructurely voided Si films due to enhanced phonon scattering across inherently voided topology, weak NP-NP and NP-substrate interface bonding within nanogranular Si films.

## 1. Introduction

Nanostructures with thermal conductivities k two or three orders of magnitude lower than those of their bulk counterparts have been recently pointed out as a new thermal insulating nanomaterials with unique thermal, mechanical and electrical properties. Low heat-conductive composite semiconducting nanoparticles (NPs) are currently used [1] and envisioned [2] as heat insulators for thermal management, thermoelectric energy conversion and optoelectronic devices. The phonon scattering in NP films can be tailored by controlling NP sizes, matrix material surrounding the NPs and substrate on which the NPs are deposited.

Colloidal NPs can be uniformly deposited on a flat substrate surface by spin coating, which requires highly concentrated NP solutions with viscous organic matrix [3], electrophoretic deposition techniques with required electrically conductive surface [4], and centrifugation [5]. Drop casting, on the other hand, is a viable alternative bottom-up technique for the simple and inexpensive fabrication of thin nanogranular solid films with limited controllability, which has the potential to become a large-scale coating method. This technique is widely used to fabricate thermoelectrics [6] and hybrid polymer insulating films [7].

Photothermal (PT) phenomena obviously depend on both the optical absorbance and heat conductivity, which are rather different in the bulk materials, thin film and nanostructures. The corresponding non-destructive PT methods are employed to heat a sample and the resulting spatial temperature distribution can be used for the assessment of k and other thermal properties. When it comes to probe thermal transport properties of NP-based films, it is sufficient to use mild laser powers in contrast to the case of bulk materials, which would require much higher laser power inputs. To study heat propagation properties in nanostructured materials, a large variety of PT techniques, such as Raman spectroscopy [8], thermoreflectance [9], spectral radiometry [10] and others have been employed.

In addition to assessment of thermal properties, the PT effects are also utilized in a variety of critical applications ranging from photothermal imaging [11], PT therapy [12] to hyperthermia for cancer therapy and theranostics [13]. In particular, porous Si (Por-Si) and Si NPs are also attractive for PT therapeutic applications due to their biocompatibility, biodegradability, high surface area and controllable pore diameter [14].

Micro-Raman spectroscopy is known to be a non-destructive and highly sensitive PT technique widely used to study various kinds of non-metallic materials including NPs via probing their phonon vibrational properties and local photo-induced T. In this technique, the laser light is used simultaneously for sample heating and for recording of temperature dependent Raman spectra [15]. Heat transport properties of bulk single crystalline Si at high temperatures, Si nanofilm on substrate [16] and thick porous Si layers [17] were studied by means of this technique. Spectral shift of a Raman peak ensured by the laser-induced temperature rise can be used to extract k of heated materials. In particular, thermally anisotropic 2D materials such as Si membranes have been extensively studied where the in-plane heat conduction is much stronger than the cross-plane one [18]. 1D heat-conductive structures, such as single Si nanowires with diameters ≤ their phonon mean free paths ($l_{MFP}$) exhibit suppressed heat conductivities due to the phonon scattering [19].

Low power CW laser-induced heating of Si NPs causes softening of the first-order Raman Si-Si transverse optical phonon mode, accompanied by a decrease in the corresponding phonon lifetimes [20], while the same Raman shift was observed for bulk crystalline Si heated at a substantially higher incident laser powers [21] because bulk Si is much more heat-conductive compared to Si NPs. Strong thermal stress causes a singlet–doublet splitting of the Raman peaks into LO and TO phonons, which leads to the phase transition provoking asymmetric broadening and spectral downshift of the Raman peak [22]. Heating of the NPs can actually be quite significant, even at mild laser irradiating powers leading to large phonon softening and spectral broadening, accompanied by the decay of optical phonon lifetime and pronounced anharmonicity of interatomic potential in the form of 3 and 4-phonon processes [23].

To the best of our knowledge, the thermal transport in drop-casted substrate-supported randomly packed Si nanogranular films with defined NP sizes and spherical shapes, has never been studied before compared to other various types of nanostructurally voided Si films ranging from low porosity pressure sintered nanostructured bulk Si (sint-Si) [24,25,26,27,28], crystalline porous Si (c-por-Si) [29,30,31,32,33,34,35,36,37,38,39,40,41,42,43,44,45,46,47,48], amorphous porous Si (a-por-Si) [49,50,51,52], crystalline porous Si nanowire (c-por-Si NW) films [53,54,55,56] to c-por-Si membranes [57,58]. As opposed to mainly top-down fabrication methods [29,30,31,32,33,34,35,36,37,38,39,40,41,42,43,44,45,46,47,48], the bottom-up approach, such as drop-casting, used in the present work, has shown to be scalable, simple and cost-effective way to produce nanogranular medium, i.e., new type of nanostructurely voided Si films.

In this paper, we report PT effects observed by means of the micro-Raman spectroscopy in Si nanogranular films with different thicknesses formed by NPs with average size of 100 ± 50 nm deposited on a silica-based glass substrate. The films are inherently voided due to the presence of air inclusions between the stacked spherical NPs. The photo-induced T growth estimated by the micro-Raman measurements was correlated with finite difference time domain (FDTD) simulation of the absorbed laser light and steady-state heat transport finite element modeling (FEM) results. This correlation procedure allowed the estimation of the thermal conductivities of our drop-casted Si nanogranular thin films that have various thicknesses. Measured k are compared with those obtained on other nanostructurely voided Si-based materials and discussed from the point of view of their potential heat insulating and thermoelectric applications.

## 2. Materials and Methods

### 2.1. Sample Preparation

The initial pure, highly crystalline Si NP powders with diameters of 100 ± 50 nm (SkySpring Nanomaterials, Houston, TX, USA) were prepared by chemical vapor deposition (CVD). Then, the powders were dispersed in distilled water to obtain colloids with different concentrations (3–30 mg/mL) and sonicated for 60 min at 50 W of sonication power at a rate of 40 kHz to avoid large particle conglomerations. In our bottom-up approach, the colloidal NPs were drop-casted on a glass substrate and dried to obtain films with various thicknesses (2–50 µm) at room T.

**Figure 1:**
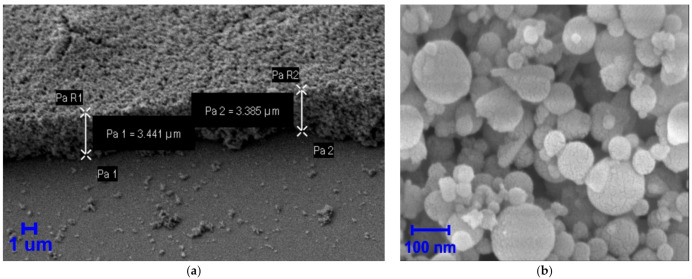

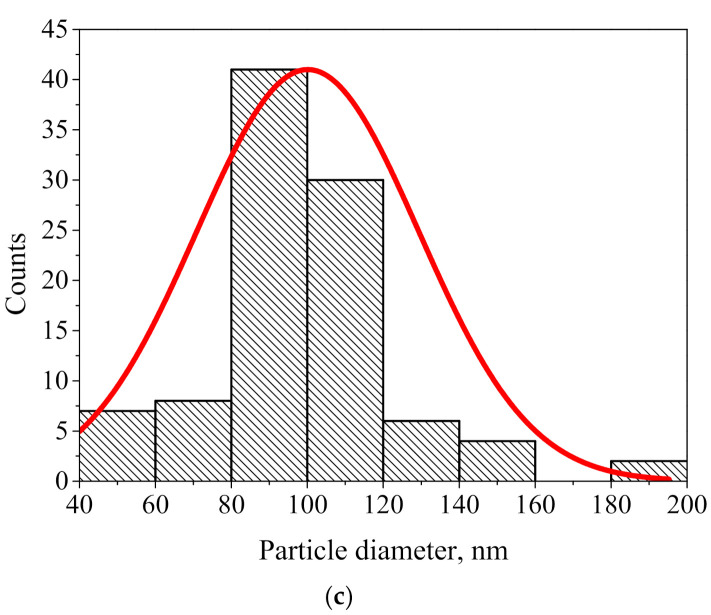
SEM images of Si nanogranular film (**a**) and a closer view of Si NPs (**b**) within a film with a typical NP size of 100 ± 50 nm (**c**).

Figure 1a presents a tilted to 45 degree cross-section image of a typical NP film obtained by scanning electron microscopy (SEM) Crossbeam 540 (Zeiss, Oberkochen, Germany) at relatively low magnification. Figure 1b shows an SEM image of a closer look on a typical part of the dried colloidal Si NPs within the formed film sample with NP sizes ranging from 50 to 150 nm. As shown in Figure 1c, the NP size distribution histogram obtained after ImageJ processing of the SEM images among counted 100 NPs.

### 2.2. Micro-Raman Spectroscopy

Laser-induced heating was implemented by CW Torus diode-pumped solid-state 532 nm single longitudinal mode laser (Laser Quantum, Stockport, Cheshire, UK). The beam was focused on Si NP film samples down to a spot of 2 μm diameter by OptoSigma microscope objective with 20× magnification and 0.5 numerical aperture. Simultaneous micro-Raman spectral measurements on the laser-heated samples were performed in real time using Nanofinder 30 confocal Raman microscope (Sol Instruments, Minsk, Belarus) (with 35 cm focal length spectrograph) coupled to iCCD camera from Andor iStar (Oxford Instruments, Abingdon, UK) operating in static mode. Diffraction grating with 1800 grooves/mm groove density yielded a spectral resolution of 1.4 cm^−1^. The pixel size on the iCCD camera and entrance slit to the spectrometer were 13 and 150 μm, respectively. All reported intensities, positions and linewidths of measured Raman peaks are the results of the Lorentzian fitting.

### 2.3. Local Temperature Measurements

Local laser-induced T was determined from measured Raman Stokes/anti-Stokes integrated intensity ratio, $I_{S}$/$I_{AS}$ [21]:

(1) $$\frac{I_{s}}{I_{AS}}=\frac{\left(\alpha_{I}+\alpha_{AS}\right)}{\left(\alpha_{I}+\alpha_{S}\right)}{\left(\frac{\omega_{S}}{\omega_{AS}}\right)}^{3}\frac{S\left(\omega_{I},\omega_{S}\right)}{S\left(\omega_{I},\omega_{AS}\right)}e^{\frac{\hbar \omega_{0}}{k_{B}T}}$$

where, $I_{S}$ and $I_{AS}$ refer to the integrated intensities of the Stokes and anti-Stokes bands at the same incident laser power. The ratio is proportional to the Boltzmann factor $e^{\frac{\hbar \omega_{0}}{k_{B}T}};\;\omega_{0}$ is the phonon frequency; $\omega_{S}$, $\omega_{AS}$ and $\omega_{I}$ are the Stokes, anti-Stokes and incident light photon frequencies, respectively; $\alpha_{S}$,$\;\alpha_{AS}$ and $\alpha_{I}$ are the optical absorption coefficients at corresponded light frequencies; $S\left(\omega_{I},\omega_{S}\right)$ and $S\left(\omega_{I},\omega_{AS}\right)$ are the Raman cross-sections of inelastically scattered photons for Stokes and anti-Stokes sides, respectively. The scattering intensities of both (Stokes and anti-Stokes) spectral peaks depend on the phonon populations of the initial states of the material, which, in turn, depend on the temperature. At thermodynamic equilibrium, the lower state will be more populated than the upper state. Therefore, the rate of transitions from the more populated lower state to the upper state (Stokes transitions) will be higher than in the opposite direction (anti-Stokes transitions).

### 2.4. FDTD Electromagnetic and FEM Heat Modeling

To describe and quantitatively estimate light absorption in our films, the Lumerical FDTD Module (Ansys, Canonsburg, PA, USA) is used to solve electromagnetic Maxwell’s equations in the time domain on a discrete spatial and temporal grid cell (Yee cell), where the field derivatives in both time and space are handled with finite differences and are second order accurate when the grid is uniform.

The fundamental simulation quantities were calculated at each cubic mesh point, which was chosen to be 7 nm. Size distribution of NPs shown in Figure 1c were taken into account in our simulation. A random localization of the NPs forming the nanogranular thin film was considered. Physical properties of Si and SiO_2_ were taken from Lumerical software material libraries (Ansys, Canonsburg, PA, USA) for Si NPs and glass substrate, respectively.

As Lumerical Heat Transfer Module uses FEM, main simulation quantities are calculated at each mesh vertex. Tetrahedral mesh size used for the 3D heat transport simulation was chosen to be in 0.1–2 µm range for the corresponding film thickness range of 2–50 µm. Since a CW laser beam was used for the sample heating, the steady state Fourier heat conduction equation was solved to find the spatial distribution of T:

(2) $$-[\frac{d}{dx}\left(k\frac{dT}{dx}\right)+\frac{d}{dy}\left(k\frac{dT}{dy}\right)+\frac{d}{dz}\left(k\frac{dT}{dz}\right)]=Q,$$

where Q is the applied heat energy density transfer rate {W/$m^{3}$} defined by the incident laser radiation heating source.

## 3. Results and Discussion

### 3.1. FDTD Modeling of Light Penetration

**Figure 2:**
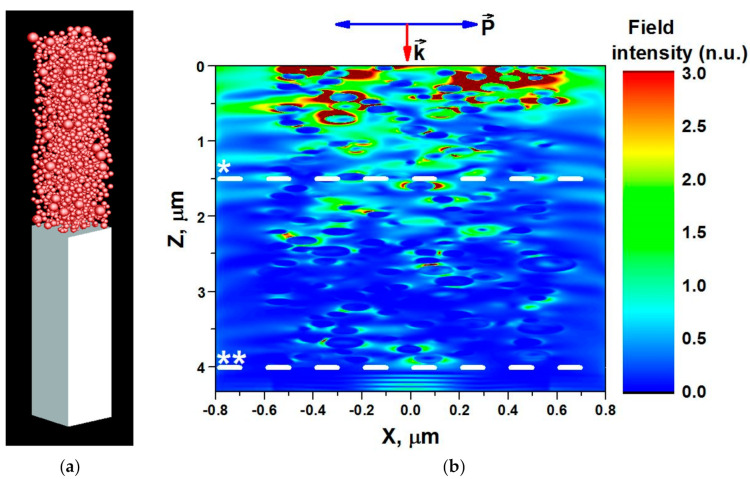
FDTD simulated (**a**) nanogranular porous Si NP film on a continuum SiO_2_ glass substrate, and (**b**) in−depth distribution of incoming light (532 nm wavelength) field intensity in normalized units (n.u.) in the nanogranular porous Si NP film (70% porosity, 4 µm depth (along z-direction) and (1.6 µm × 1.6 µm) lateral area within xy-plane with the laser beam diameter (2 µm) fitting into this lateral area). The incident laser beam is shown with wave-vector $\vec{k}$ and polarization $\vec{P}$. * absorption depth of the incoming light (532 nm), ** porous Si NP film/silica glass substrate interface.

When nanogranular films are illuminated, some incident photons are scattered by Si NPs while others are absorbed, and the both processes contribute to the extinction coefficient [59]; NP-added structures show an excellent absorption performance [60]. The laser light penetration simulated by electromagnetic FDTD across 4 µm-thick Si NP film with 1 µm × 1 µm lateral surface area on a 4 µm thick silica glass substrate is shown in Figure 2a. Size distribution of Si NPs forming the voided film and corresponding to the data shown in Figure 1 were taken into account. The morphological structure of simulated Si nanogranular film was based on the SEM image processing performed by the ImageJ software with estimated porosity of 0.7, SEM images before and after ImageJ processing are shown in Figure S1 of Supplementary Materials. The Si NP film/silica substrate is considered to be illuminated by an electromagnetic plane wave with 532 nm wavelength polarized in the direction ($\vec{P}$) parallel to the Si NP film/silica interface. The plane wave with the square of 1.6 µm × 1.6 µm propagates in the direction of light wavevector $\vec{k}$ perpendicular to the Si NP film/silica interface.

Figure 2b shows an example of the spatial distribution of the normalized incoming light intensity ($E^{2}/E_{0}^{2}$), where E is the magnitude of local transmitted electric field and $E_{0}$ is the magnitude of the incoming electric field along the depth of the Si NP film. Due to light absorption by Si NPs, the incoming light field intensity decays exponentially along the z-axis. The absorption depth (indicated by *-level in Figure 2b) at which the incident light intensity was reduced by factor of *e* was estimated to be about 1.5 µm. This estimation is confirmed by in-depth distribution of the absorbed power density (see Figure S2 in Supplementary Information), which is mainly concentrated in the near surface region. The light field intensity is much higher inside air voids (scattered light) than within the NPs.

### 3.2. Laser-Induced Phase Transition in Si Nanogranular Films

Laser-induced heating has a significant effect on phase transformation in Si nanostructures [22,61]. At room temperature, the cubic phase (c-Si) exhibits diamond-like cubic structure where each Si atom is distant from its four nearest neighbors at 2.73 Å.

At a threshold laser power, resulting in a maximal thermally induced stress, a photo-induced structural relaxation in the overheated part of Si nanostructures leads to the formation of the new hexagonal (hex-Si) phase characterized by Raman bands centered near 504 and 497 cm^−1^. These bands differ from the well-known cubic lattice with the Raman peak at 520 cm^−1^ at room temperature. This phase transition is supposed to be not purely thermally induced but also photo-induced. The formation of the hex-Si phase is accompanied by the absorption of electromagnetic radiation and a reduction in the overall mechanical stresses and, consequently, by partial quenching of the splitting between LO and TO phonon modes [22].

**Figure 3:**
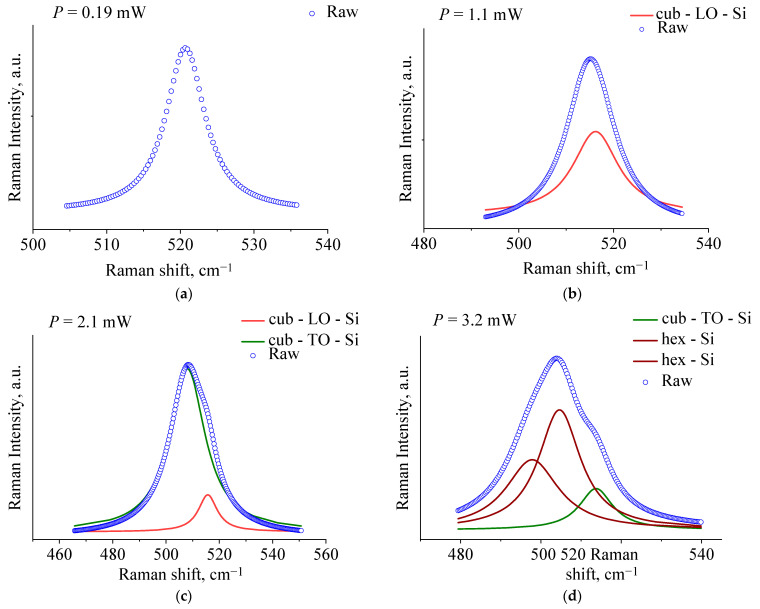
Deconvoluted Raman spectra of the films formed by Si NPs with average size of 100 nm demonstrating a photo-induced transition of phase from cubic (**a**–**c**) to hexagonal one (**d**).

Figure 3 illustrates Lorentzian fitted Raman spectra of our Si NP films. The Raman bands obtained at laser power of 2.1 mW and centered near 515 and 507 cm^−1^ correspond, respectively, to the LO and TO phonon modes of cubic c-Si under photo-thermal and mechanical stresses caused by a temperature gradient through Si NP films. At the threshold laser power of 3.2 mW, the photo-induced structural relaxation in the overheated part of Si NPs leads to the formation of the hexagonal (hex-Si) phase characterized by Raman bands centered at 504 and 497 cm^−1^.

### 3.3. Laser Induced Heating and Thermal Conductivity of Si Nanogranular Films

**Figure 4:**
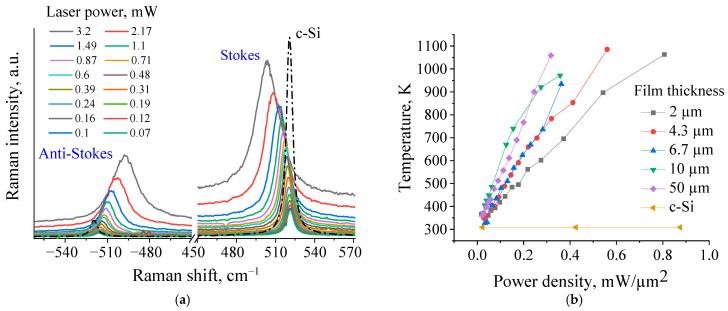
(**a**) Measured Stokes and ant-Stokes Raman spectra at various laser powers and (**b**) corresponding laser induced temperature rises in the Si NP films with various thicknesses and a bulk crystalline silicon substrate (c-Si) versus laser power densities.

As seen from Figure 4a, the main Raman peak of the heated Si nanogranular films attributable to the cubic Si LO phonon mode at ~519 cm^−1^ shifts towards lower phonon frequencies when the laser power increases. The frequency shifts of the Stokes and anti-Stokes peaks are symmetric with respect to the Rayleigh line because they correspond to the energy difference between the same upper and lower resonant states [62]. A laser heating-induced red-shift of the Raman peak as high as 20 cm^−1^ over the 0.07–3.2 mW range of the incident laser powers is also accompanied by a characteristic spectral line broadening.

Figure 4b illustrates laser-induced T evolution for the films of different thicknesses. The thinner films are less heated than the thicker ones due to more efficient heat sinking toward the silica glass substrates on which the films are deposited. The similar substrate effect was also earlier observed for polycrystalline Si discs on fused silica and sapphire substrates [63]. Thus, an appreciable amount of laser-induced heat will easily “sink” from the thinnest 2 µm-thick film toward the bulk glass substrate. At film thicknesses >2 µm, the effect of the heat-sinking to the substrate becomes weaker and leads to the higher temperature excursions within the films. We observed the expected T increase with the rise in incident laser power for all film thicknesses. The laser-induced local heating also resulted in the enhancement of thermal radiation background, as evidenced from the more significant rise in the baseline on the anti-Stokes side compared to the Stokes side of the recorded Raman spectra as shown in Figure 4a.

The temperature-induced effect on vibrational states in bulk c-Si and Si NP films can be estimated from the Raman peak’s position and linewidth. Indeed, a higher anharmonicity in the vibrational potential energy for the Si NP films can be noticed in comparison with bulk c-Si irradiated at the same laser powers. Because of anharmonicity of the lattice forces, the incident photons can interchange energy with the phonon lattice modes and therefore maintain thermal equilibrium. Additionally, an increase in the interatomic lattice parameter leads to the corresponding bonds weakening. The spectral linewidth scales reciprocally with the lifetime of the optical phonon mode decay processes. The thermal interaction increases with the temperature rise, causing the suppression of mean phonon free path ($l_{MFP}$) with a corresponding decay in phonon lifetime [21,64].

The heat conduction through a Si NP film deposited on 1 mm-thick silica substrate with surface area of 100 µm × 100 µm was numerically modeled by 3D Heat Transport Lumerical FEM Solver. The local T values estimated from micro-Raman spectroscopy and achieved inside a near-surface cylindrical region of the laser spot with 2 µm diameter and 1.5 µm optical penetration depth in Si NP films of various film thicknesses (2–50 µm) were fitted by the FEM. Heat flows across the films are deduced from the Raman measurements in Figure 4b to remain constant at *T* < 700 K. The dependence of the heat flux on T is expected to be nonlinear at *T* > 700 K. This could be due to: (i) possible T-dependent structural variations in the films as well as (ii) T-dependent thermal conductivities of the Si NPs. Thus, the FEM modeling of the laser-induced heat conduction is performed at *T* < 700 K to avoid any non-linear behavior between incident powers and resulting T.

**Figure 5:**
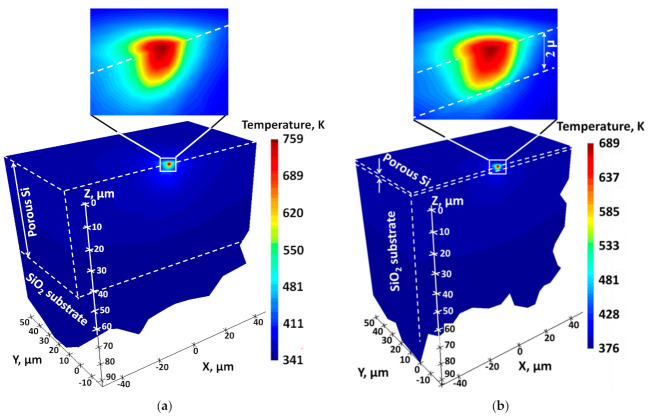
In-depth T distribution for porous Si NP films deposited on 1 mm thick SiO_2_ glass substrate with the film thickness of (**a**) 50 µm and (**b**) 2 µm. Film heating is induced by a 532 nm wavelength laser beam of 2 µm diameter focused on the film surface.

The maximum T values measured by the Raman technique and determined along the optical penetration depth of 1.5 µm for 532 nm laser beam were fitted for 50 and 2 µm-thick Si nanogranular films, as shown in Figure 5a,b, respectively. The single fitting parameter allowing a precise correlation between the experimentally measured and theoretically calculated T values was k of the films. The resulting 3D T distributions are also numerically predicted for each case. For the thermally thick substrate shown in Figure 5a, the film thickness (50 µm) being much larger than the laser beam diameter (2 µm) results in the almost perfect semispherical T isotherms. This implies that the laser beam focused at the sample surface mimics a point heating source. The T gradient is mainly localized within the subsurface depth of 5 µm. In contrast, as shown in Figure 5b, for thermally thin Si nanogranular films with thickness of 2 µm, the T profiles are strongly affected by the close proximity of the film/substrate interface.

**Figure 6:**
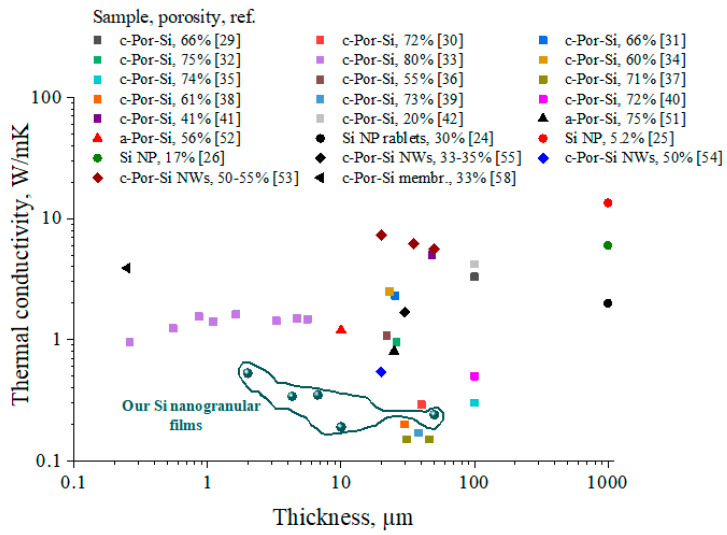
Measured thermal conductivity values of our Si nanogranular films with 70% porosity in comparison with other types of nanostructurally voided Si films (taken from the literature) versus different film thicknesses.

Taking into account geometry of the Si NP film/silica structure and of the laser-induced hot spot at the film surface as well as absorbed laser power value, one can fit the T values reached in the heated spots by k values of the Si NP films. Determined k values in our Si nanogranular films as a function of film thicknesses are given in Figure 6 and they are compared with those reported earlier for other types of nanostructurely voided Si films. In our films, the thermal conductivity decreases with the rising film thickness, except for the 50 μm-thick film, where Si NPs are assumed to be more tightly packed. Packing results in the increase in the neck areas between interconnected Si NPs and a global reduction in the effective film porosity. The thin layers are more thermally conductive than the thick ones, because the former are more affected by the heat-sinking glass substrate. Additionally, with the reduction in the film thickness, the in-plane heat conduction contribution becomes substantial compared to the cross-plane k. Therefore, it is essential to compare heat conduction in various nanostructurely voided Si materials as function of their thicknesses. Moreover, as can be seen from Figure 6 and Supplementary Table S1, the k can be controlled by means of different etching techniques leading to formation of various porous morphologies. For example, n-type thick porous Si samples have multi-porous (nano, meso, micro) morphologies, while p+, p-type samples have single porous morphologies [30]. In general, measured k = 0.19–0.53 W/mK of our Si nanogranular films are in the range of the lowest k values of nanostructurely voided Si films [29,30,31,32,33,34,35,36,37,38,39,40,41,42,43,44,45,46,47,48], Si nanowire films [53,54,55,56], amorphous porous Si films [49,50,51,52], crystalline porous Si membranes [57,58] and sin-Si NP tablets [24,25,26,27,28].

Additionally, when the size of a nanostructure ≤ $l_{MFP}$, phonons collide with intergranular boundaries much more often than in single crystals. This additional phonon scattering mechanism suppresses heat flow between NPs and thus reduces the effective k of Si NP films compared to that of the bulk c-por-Si [65,66]. $l_{MFP}$ decreasing with the particle size, leads to the reduction in the effective k [67]. Finally, a long exposure of Si NPs to an ambient-air environment can result in their increased surface oxidation causing the formation of SiO_2_ shells and leading to an additional decrease in their k [68].

## 4. Conclusions

We have investigated the PT effect and heat-conductive properties of drop-casted Si nanogranular films by means of the micro-Raman spectroscopy correlated with FDTD optical and FEM heat conduction simulations. The observed photo-induced Raman spectral variations were attributed to the cubic-to-hexagonal structural phase transition. The k in the prepared nanogranular Si films was found to be in the range of the lowest k values of nanostructurely voided Si films including bulk and thin film crystalline and amorphous porous Si structures of various porosity. It was established that: (i) presence of air voids, (ii) additional interface thermal resistance across NP-NP boundaries in our nanogranular Si films, as well as (iii) NP sizes smaller than mean phonon free path ($l_{MFP}$) ensure k values of the films which are much lower than those of the bulk c-Si.

Heating of Si nanogranular films is also heavily influenced by their thicknesses and the thermal conductive properties of the heat-sinking glass substrate on which the films were deposited, suggesting a major role played by the NP-substrate binding. Additionally, glass substrate leads to the increase in thinner films’ k, which decreases with the rising thickness until the substrate effect is negligible, which is similar to the case of porous Si films taking into account interface thermal resistance. Tailoring the film thickness, NP size and film porosity within the surrounding air matrix by bottom-up drop-casting approach opens up an avenue for effective control of heat insulating and thermoelectric performance across a variety of energy conversion applications of semiconductor NPs.

## Data Availability

Data is contained within the article and supplementary materials.

## Supplementary Materials

The following are available online at https://www.mdpi.com/article/10.3390/nano11092379/s1. Figure S1: SEM images before and after ImageJ processing for porosity estimation. Figure S2: Absorbed power density (in W/m^3^, ×10^15^) at different depths (z) of 4 µm thick porous Si NPs film on 4 µm thick glass substrate: (a) z = 0.1 µm, (b) z = 1 µm, (c) z = 2 µm, (d) z = 3 µm. Table S1: Room temperature thermal conductivity values of nanostructurely voided Si.
