# Supplementary material for: Photothermal Effects and Heat Conduction in Nanogranular Silicon Films
Source: Nanomaterials (Basel). 2021 Sep 13;11(9):2379. doi: 10.3390/nano11092379 (PMC8464803; doi:10.3390/nano11092379)
Supplement: Supplementary file 1 [file nanomaterials-11-02379-s001.zip › nanomaterials-1362925-supplementary.pdf]

Supplementary Materials

Photothermal Effects and Heat Conduction in Nanogranular Silicon Films

Bayan A. Kurbanova [1], Gauhar K. Mussabek [2,3], Viktor Y. Timoshenko [3,4,5], Vladimir Lysenko [3,6] and Zhandos N. Utegulov [1,*]

1 Department of Physics, School of Sciences and Humanities, Nazarbayev University, Nur-Sultan 010000, Kazakhstan; bayan.kurbanova@nu.edu.kz, zhutegulov@nu.edu.kz
2 Faculty of Physics and Technology, Al-Farabi Kazakh National University, Almaty 050040, Kazakhstan; gauhar.musabek@kaznu.kz
3 Laboratory "Nanotheranostics", Institute of Engineering Physics for Biomedicine, National Research
Nuclear University "MEPhI", 115409 Moscow, Russia; victor_timoshenk@mail.ru (V.Y.T.); vladimir.lysenko@univ-lyon1.fr (V.L.)
4 Faculty of Physics, Lomonosov Moscow State University, 119911 Moscow, Russia
5 Lebedev Physical Institute of RAS, 119911 Moscow, Russia
6 Light Matter Institute, UMR-5306, Claude Bernard University of Lyon, 2 rue Victor Grignard,
69622 Villeurbanne, France
* Correspondence: zhutegulov@nu.edu.kz

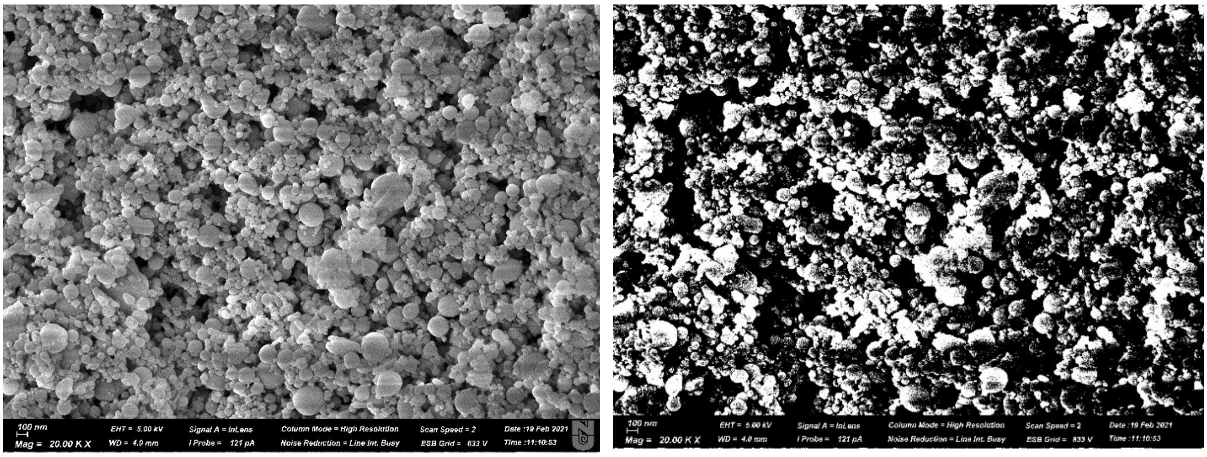

Figure S1. SEM images before (left) and after (right) ImageJ processing for porosity estimation.

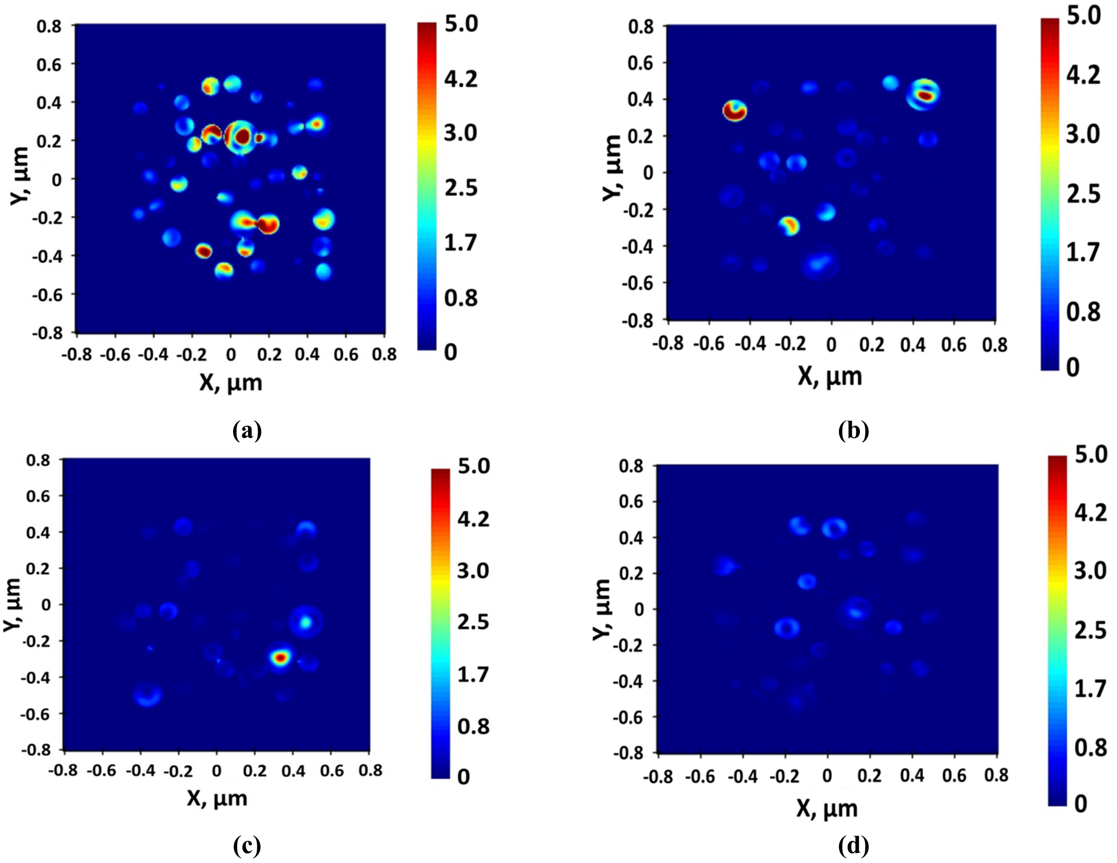

Figure S2. Maps of the absorbed power density (in W/m^3, $\times 10^{15}$) at different depths (z) of 4 µm thick porous Si NPs film on 4 µm thick glass substrate: (a) z = 0.1 µm, (b) z = 1 µm, (c) z = 2 µm, (d) z = 3 µm.

Table S1. Room temperature thermal conductivity values of various *nanostructurely voided Si.*

Photoacoustic method = PA, Phonon hydrodynamics calculations = PH, Monte Carlo simulation = MC, Volkein's DC method = VDC; Optical pump-probe technique = OPP; Lock-in thermography = LT; Scanning thermal microscopy = SThM; Molecular Dynamics Simulation = MD; Finite element mesh = FEM; Electrothermal response = ETR; Laser flash method = LF; Born-von Karman model = BK; Effective medium model = EM; Electron beam heating method = EBH; Two-laser Raman thermography = TLRT;

Porous Si layer thickness (micron)	Porosity, %	Thermal conductivity (W/m K)	Measurement/ calculation technique	Sample description	Reference
			**Porous Si**		
10	50	3.9	PA	c-Por-Si, p+ type, anodization-etched	[34]
23	60	2.5	PA	c-Por-Si, p+ type, anodization-etched	[34]

75	40	31.2	PA	c-Por-Si, n- type, electrochemical anodic-etched	[34]
180	40	31.2	PA	c-Por-Si, n- type, d = 200-500 nm, electrochemical anodic-etched	[34]
100	66	3.3	3ω	c-Por-Si, n type, d = 47nm(pore diameter), electrochemical etched	[29]
100	43	3.7	3ω	c-Por-Si, n type, d=27nm(pore diameter), electrochemical etched	[29]
100	40	5.5	3ω	c-Por-Si, n type, d=35nm(pore diameter), electrochemical etched	[29]
100	33	5	3ω	c-Por-Si, n type, d=32nm(pore diameter), electrochemical etched	[29]
100	17	13.7	3ω	c-Por-Si, n type, d=64nm(pore diameter), electrochemical etched	[29]
100	60	17.6	3ω	c-Por-Si, n type, d=235nm(pore diameter), electrochemical etched	[29]
100	42	24	3ω	c-Por-Si, n type, electrochemical etched	[29]
-	40	1	PH	c-Por-Si	[44]
75	40	29.6	PH	c-Por-Si, p+ type, anodization-etched	[44]
10	50	5.9	PH	c-Por-Si, p+ type, anodization-etched	[44]
23	60	4	PH	c-Por-Si, p+ type, anodization-etched	[44]
31	64	0.29	PH	c-Por-Si, p-type, boron-doped, electrochemical etched	[44]
46	71	0.16	PH	c-Por-Si, p-type, boron-doped, electrochemical etched	[44]
31	79	0.10	PH	c-Por-Si, p-type, boron-doped, electrochemical etched	[44]
31	89	0.02	PH	c-Por-Si, p-type, boron-doped, electrochemical etched	[44]
0.25	15.9	21.9	TLRT	c-Si membrane, d = 135 nm(pore diameter)	[58]
0.25	24.6	8.5	TLRT	c-Si membrane,d = 135 nm(pore diameter)	[58]
0.25	33.2	3.9	TLRT	c-Si membrane,d = 135 nm(pore diameter)	[58]
310	71	0.15	3ω	c-Por-Si, p-type, boron-doped, electrochemical etched, on si substrate	[37]

46	71	0.15	3ω	c-Por-Si, p-type, boron-doped, electrochemical etched, on si substrate	[37]
31	64	0.20	3ω	c-Por-Si, p-type, boron-doped, electrochemical etched, on si substrate	[37]
31	79	0.04	3ω	c-Por-Si, p-type, boron-doped, electrochemical etched, on si substrate	[37]
31	89	0.03	3ω	c-Por-Si, p+-type, high-doped, electrochemical etched, on si substrate	[37]
0.05	30	5.5	MC	c-Por-Si, p+ type, electrochemical etched	[45]
0.15	30	5.6	MC	c-Por-Si, p+ type, electrochemical etched	[45]
0.26	30	6.2	MC	c-Por-Si, p+ type, electrochemical etched	[45]
0.5	30	6.4	MC	c-Por-Si, p+ type, electrochemical etched	[45]
1	30	6.5	MC	c-Por-Si, p+ type, electrochemical etched	[45]
45	40	2.93	PA	c-Por-Si, macro, micro, nano mixed porous, n type, el.etched	[30]
60	57	1.03	PA	c-Por-Si, macro, micro, nano mixed porous, n type, el.etched	[30]
40	72	0.29	PA	c-Por-Si, macro, micro, nano mixed porous, n type, el.etched	[30]
4.84	0.23	0.55	VDC	d = 10.9(pore diameter), n type PS	[46]
4.45	0.23	0.55	VDC	d = 10.9(pore diameter), n type PS	[46]
4.49	0.26	0.39	VDC	d = 2.3(pore diameter), n type PS	[46]
4.57	0.26	0.4	VDC	d = 2.3(pore diameter), n type PS	[46]
23	60	6.1	PH	R = 10(pore radius), c-Por-Si, p+ type, anodization-etched	[47]
31	64	2.17	PH	R = 2(pore radius), c-Por-Si, p-type, boron-doped, electrochemical etched, on si substrate	[47]
46	71	1.56	PH	R = 2(pore radius), c-Por-Si, p-type, boron-doped, electrochemical etched, on si substrate	[47]
31	79	0.99	PH	R = 3(pore radius), c-Por-Si, p-type, boron-doped, electrochemical etched, on si substrate	[47]
31	89	0.18	PH	R = 5(pore radius), c-Por-Si, p-type, boron-doped,	[47]

				electrochemical etched, on si substrate	
19	61	0.15	OPP	c-Por-Si, p type, electrochemical etched	[39]
27	66	0.13	OPP	c-Por-Si, p type, electrochemical etched	[39]
38	73	0.17	OPP	c-Por-Si, p type, electrochemical etched	[39]
3.2	27.5	20.8	LT	c-Por-Si, d=100nm, p-type, boron doped, etched-sintered	[31]
20.8	37.2	12.7	LT	c-Por-Si, d=100nm, p-type, boron doped, etched-sintered	[31]
20.7	44.2	11.3	LT	c-Por-Si, d=100nm, p-type, boron doped, etched-sintered	[31]
25.1	48.4	8.7	LT	c-Por-Si, d=100nm, p-type, boron doped, etched-sintered	[31]
26.8	51.7	6.1	LT	c-Por-Si, d=100nm, p-type, boron doped, etched-sintered	[31]
26.9	58.8	4.5	LT	c-Por-Si, d=100nm, p-type, boron doped, etched-sintered	[31]
25.5	66.2	2.3	LT	c-Por-Si, d=100nm, p-type, boron doped, etched-sintered	[31]
26	55	1.93	PA	c-Por-Si, p-type, nitrided, KOH etched	[32]
26	55	1.12	PA	c-Por-Si, p-type, anodized, KOH etched	[32]
26	75	0.74	PA	c-Por-Si, p-type, nitrided, KOH etched	[32]
26	75	0.95	PA	c-Por-Si, p-type, anodized, KOH etched	[32]
0.26076	80	0.95013	SThM	c-Por-Si, p+-type, boron doped, electrochemical dissolution	[33]
0.55281	80	1.23833	SThM	c-Por-Si, p+-type, boron doped, electrochemical dissolution	[33]
0.86159	80	1.55207	SThM	c-Por-Si, p+-type, boron doped, electrochemical dissolution	[33]
1.10155	80	1.40869	SThM	c-Por-Si, p+-type, boron doped, electrochemical dissolution	[33]
1.63169	80	1.61738	SThM	c-Por-Si, p+-type, boron doped, electrochemical dissolution	[33]
3.27791	80	1.43424	SThM	c-Por-Si, p+-type, boron doped, electrochemical dissolution	[33]
4.66557	80	1.49954	SThM	c-Por-Si, p+-type, boron doped, electrochemical dissolution	[33]
5.62541	80	1.47399	SThM	c-Por-Si, p+-type, boron doped, electrochemical dissolution	[33]

0.26076	80	0.95013	SThM	c-Por-Si, p+-type, boron doped, electrochemical dissolution	[33]
100	74	0.3	Raman	c-Por-Si, p+-type, mesoporous, anodic dissolution process	[35]
0.054	15	3.91	MD	Bulk c-Si matrix, simulation cell length 24 – 136nm, D(pore) = 3nm, N(pore number) = 99	[57]
0.081	15	4.26	MD	Bulk c-Si matrix, simulation cell length 24 – 136nm, D(pore) = 3nm, N(pore number) = 153	[57]
0.1	15	4.67	MD	Bulk c-Si matrix, simulation cell length 24 – 136nm, D(pore) = 3nm, N(pore number) = 198	[57]
0.135	15	4.77	MD	Bulk c-Si matrix, simulation cell length 24 – 136nm, D(pore) = 3nm, N (pore number)= 252	[57]
0.054	25	2.12	MD	Bulk c-Si matrix, simulation cell length 24 – 136nm, D(pore) = 3.5nm, N(pore number) = 99	[57]
0.081	25	2.26	MD	Bulk c-Si matrix, simulation cell length 24 – 136nm, D(pore) = 3.5nm, N(pore number) = 153	[57]
0.1	25	2.37	MD	Bulk c-Si matrix, simulation cell length 24 – 136nm, D(pore) = 3.5nm, N(pore number) = 198	[57]
0.135	25	2.48	MD	Bulk c-Si matrix, simulation cell length 24 – 136nm, D(pore) = 3.5nm, N(pore number) = 252	[57]
0.054	35	1.09	MD	Bulk c-Si matrix, simulation cell length 24 – 136nm, D(pore) = 3.9nm, N(pore number) = 99	[57]
0.081	35	1.28	MD	Bulk c-Si matrix, simulation cell length 24 – 136nm, D(pore) = 3.9nm, N (pore number)= 153	[57]
0.1	35	1.20	MD	Bulk c-Si matrix, simulation cell length 24 – 136nm, D(pore) = 3.9nm, N(pore number) = 198	[57]
0.135	35	1.31	MD	Bulk c-Si matrix, simulation cell length 24 – 136nm, D(pore) = 3.9nm, N(pore number) = 252	[57]

0.054	45	0.65	MD	Bulk c-Si matrix, simulation cell length 24 – 136nm, D(pore) = 4.3nm, N(pore number) = 99	[57]
0.081	45	0.73	MD	Bulk c-Si matrix, simulation cell length 24 – 136nm, D(pore) = 4.3nm, N(pore number) = 153	[57]
0.1	45	0.70	MD	Bulk c-Si matrix, simulation cell length 24 – 136nm, D(pore) = 4.3nm, N(pore number) = 198	[57]
0.135	45	0.75	MD	Bulk c-Si matrix, simulation cell length 24 – 136nm, D(pore) = 4.3nm, N(pore number) = 252	[57]
bulk	8	1.5	MD	6x6x6 contained unit cell, side length 3.258nm, D(pore) = 1.74nm	[48]
bulk	15	2.5	MD	6x6x6 contained unit cell, side length 3.258nm, D(pore) = 1.74nm D(pore) = 2.17nm	[48]
bulk	27	4.5	MD	6x6x6 contained unit cell, side length 3.258nm, D(pore) = 1.74nm D(pore) = 2.61nm	[48]
bulk	38	9	MD	6x6x6 contained unit cell, side length 3.258nm, D(pore) = 1.74nm D(pore) = 2.93nm	[48]
22	55	1.08	3ω	c-Por-Si, p-type, electrochemical anodization	[36]
25	75	0.8	SThM	a-P0r-Si, p+-type, electrochemical etched	[51]
100	72	0.5(3w)	3ω	c-Por-Si, p+-type, electrochemical etched	[40]
48	41	5	FEM, PT	c-Por-Si, n-type, electrochemical etched	[41]
10	56	1.2	Raman, SThM	a-PSi, p+-type, electrochemical etched	[52]
100	20	4.2	ETR, FEM	c-Por-Si, n-type, anodization	[42]
50	45	2.3	PA	c-Por-Si, p-type, electrochemical etched	[53]
50	55	1.8	PA	c-Por-Si, p-type, electrochemical etched	[53]
50	65	1.4	PA	c-Por-Si, p-type, electrochemical etched	[53]
26.6	23	1.2	PA	c-Por-Si, p-type, electrochemical anodic etched	[38]

27.2	37	0.3	PA	c-Por-Si, p-type, electrochemical anodic etched	[38]
28.3	52	0.25	PA	c-Por-Si, p-type, electrochemical anodic etched	[38]
30	61	0.2	PA	c-Por-Si, p-type, electrochemical anodic etched	[38]
**Si NP tablets**					
bulk	30	1.5	LF	c-Si NP, D_{NP} =5.6nm, Spark plasma sintered	[24]
bulk	30	2	LF	c-Si NP, D_{NP} =60nm, Spark plasma sintered	[24]
bulk	9	25.7	BK	c-Si NP, D_{NP} = 70nm, Spark plasma sintered	[25]
bulk	4.8	21.8	BKl	c-Si NP, D_{NP} = 60nm, Spark plasma sintered	[25]
bulk	5.2	13.5	BK	c-Si NP, D_{NP} = 40nm, Spark plasma sintered	[25]
bulk		10	LF	c-Si NG, D_{NP} = 5-20nm, dc hot pressed.	[27]
bulk		10	Calculated from thermal diffusivity	c-Si NP, D_{NP} = 5-1-nm, ball milled and sintered	[28]
bulk	17	6	3ω	Si NC, D_{NP} = 64nm	[26]
**Si NW films**					
30	33 - 35	1.68	Comsol simulation	c-Por-Si NWs, D_{NW} = 20-200nm, met.assist.chem.etched	[55]
20	50	0.29	Raman-Fourier	D(beam) = 1 micron, c-Si NW, 50%, d=150nm, p-type,met.assist.chem.etched	[54]
35	50	0.25	Raman-Fourier	D(beam) = 1 micron, c-Si NW, 50%, d=150nm, p-type,met.assist.chem.etched	[54]
20	50	0.54	Raman-Fourier	D(beam) = 2 micron, c-Si NW, 50%, d=150nm, p-type,met.assist.chem.etched	[54]
35	50	0.36	Raman-Fourier	D(beam) = 2 micron, c-Si NW, 50%, d=150nm, p-type, met.assist.chem.etched	[54]
0.05	43	0.33	EBH	c-Por-Si NWs , D_{NW} = 8nm, etched	[56]
20	50-55	7.3	PA	c-Por-Si NWs, D_{NW} =100nm, p-type, met.assis.chemical etched	[53]
35	50-55	6.2	PA	c-Por-Si NWs, D_{NW} =100nm, p-type, met.assis.chemical etched	[53]
50	50-55	5.6	PA	c-Por-Si NWs, D_{NW} =100nm, p-type, met.assis.chemical etched	[53]
